# Supplementary material for: The yellow gene influences Drosophila male mating success through sex comb melanization
Source: eLife. 2019 Oct 15;8:e49388. doi: 10.7554/eLife.49388 (PMC6794089; doi:10.7554/eLife.49388)
Supplement: Supplementary file 1. [file elife-49388-supp1.pdf]

IDT gene blocks for cloning 42D04 dsx enhancer sub-fragments into pBPGUw (Supplementary Figure S6B)

42D04_A-GAL4 (5'-3') Gibson Tails for cloning into pBPGUw digested with FseI and AatII

AAAAGTGCCACCTGACGTGTTCGCGTTGCCCGGCGGTCTGCTCCAAGCCGGGATTAT
ATGCGCCCGAAATGGGGCTTAATTATATGGCCAGAATCTATCCAGGAACAGACCAC
AAAAAATACTTTTCCGCACGAACAATAAAAATATGGTTTTCTCTTCTCGCAATTAAA
GCCGTTTTTACGAGCAGAAAATTAGTGTACGAGTAGTCGCAGCTCGCGATCTGGCCT
TTGAATGGGTCTAGAATGGCTCTAAACACGCGGCAGCTGCAGCGGATAAAGGCATA
GGGCTAATAAGCTCAGGCCTTCCTTGCTGGCGATATTTACAACCTGCCAAAGTCATT
TGCATCGGCGTACAGGCATAATCAGCTTCCATCCAGAAGGCATCAATTATAGGGCTC
TCACCGAAGCCCAACCCCCTTTTTTTAAGCCAGGTTCTAAGCCCCGATGTATTCACTT
GAGCTGCATCCTCGAAATGCGGGCCATTAGGCTTCGCACTCTTTTTTTTTTGTTTTTT
TTGTTTTTGACAAGTCGAAAGCTTGTGAATCCGTGAAAACGTACTGCCCAAAAAACT
AAATTACCCTGTGTGAAGATTCAACGGCTTTTTAATTGAGTAAAATTATGGGGTACG
AACAGATTTCAACAAAAAAGATGCAATGCAAGCCGAAACACACACGTTATCTGTTA
TAAGTTATTGTTTGGCATCAATAAAAAATTTCAAAATGTTCAAACAAAAAAGGAATA
GTTTTTTTATGTTTATAATTCAGTTAATTCAGATTATGGAGTTACTGTTGGCCTTCCA
CCATTCGACTGCCAAAATATTCTCTCCGTGTAGCTATAAAAAAATATCCCGAAGCTT
GACCAAATAAGGCCCGCTAAGGCTAAAAACACTTAAACAATTAGGAAACAGAGTTA
TTTATTTAAAGGAGGGCATTGGCTGTAAGGAAGACATTTTCGATATCATTAATGTAA
TGTTAATACGGGCATTCCGTTGACAAGATCAACAGCAGGGGGAAGGAGGATGATTG
ATGCCCTGGCCCTGCTGCTTTTGTTTGTGCGATCAAAATAATTGCTCCTTCGGGCACG
ATTCTTTGCCACTCAATTTATTAAATTTATAAAATATTTATGAAGCCGTCTCTGAGTA
CCTTTACTTATAAAAACTTGTTGCTGCAAATGATGTAATTGCATGCGAAGCACTGCT
TAAAACAACAATGTTGGACCCCCCGAAAGATTGGGGCGCTGGCTCTCTGTTCCTCTG
TTGCCACTTAGTAATCGGACGCCGTCTCCGCTTGGCTTCGACTTCAGAACTGTTTACA
TTTCCACAGACACGCAATTTATGATCCGGCTTGGGGCAGGCATTGTTCGCAGCATCG
GCATCGCTGCTTGCAACTGGCAACTGGCAACCTGCAACTTCCCTGCGGCAAATACAA
TACGACTGCAAGTGCGCGAAGTGAGCGCATTCGAAACGAACTGGAATTTGGGAATG
GGCAGAGCAAAGCACTTGCAGATTTATGGCAGGCATCTGCCCAAAGGTGCCAAGAA
CTTGCTTCATAAACCGAGTGTGTGATTGGTCTTGCCGCGGGCCAAATTGATTTATGG
TCCTCTGCGGATACGTGTTAGCAGTTATCAACTGCTAAATCAGAAGAGTTTGCGCCC
GCAAATGATATTTGCCTCGTGGAATTGATGTCCCAATCTATGATTTCTAAGCTTGGTG
GTTTAATTATAACAATAAATAACAATGCACTGCAGATGGGGAGCTTAAACTGTTTTA
AAACGCTTACAGATGGCAAAAAAGTGCGTTTCACACGAAGTGGGCTTAATTGATTTC
TGCGTTCTGTAAATAATGCATGATCTATAAAACTGGGTGCTCAATTATAATAACTAA
TAGCTAGTCAGTGCTACTAAATATATATTAGAGTGCTTTAATCTGATGGCAAGTAGG
GAATCAGTGCATTTCACACGAAGATGGTGGGTCTGCCGGCCGAGCTCGCCCGG

42D04_B-GAL4 (5'-3') Gibson Tails for cloning into pBPGUw digested with FseI and AatII

AAAAGTGCCACCTGACGTCGGCGTTCCCAAGAACTTAGAATGGCAATACCTTAGGG
TAGCAGATATAAAAGCTGATTAGCAGCTGGTTGCCGATTCCGTCTGGGGGTGTGGAC

TCCGCCGAATCAGGTGCAATGAGGGCCATCCAACGCTCGGCTGACCACGTTCCCTAA
TTGCATCGCGGCACACAATGCGGCATACAAATGCAGCACAAACTACGACTACGACT
ACGGACAATTCCAAGACGCTCTGTCAAGCCAATCCAACATGACCATCCATCAAAGT
GCTAAGCAGGCATGAAACTTTTTCACAGTTTCATTTTCCACTCGCCGATGTCTTCTTT
ATGCATACGAGCATTATCATTATGGGCGCGTAATGGCAGCCAGACATGGATCAGAC
ATTACGGCGATCGTTTAGCAGAAGTGCAGACAATACAAACCGACGTCTGTCAACTG
CAGGCAGTTCGTCAATCAATTGAACACCGCTCCACCTCTCCCACCTCACCTAGCGCC
TCGAGCTCCTCAGTTGCCGTCGCTGGCAGCCAAGTAGCCAGGTTGTGGCCCCGATGT
CTGCAAACATGTAAACCCAAACGTAATCTCGATCGCCGCCACGTAGCTAACTAACCC
GTCTTCAAACAGGAACCGAGATCGGGTTTCCAGCCGGCCATGGCAACTTTGTTGCAA
ATTTCCTAGTCGCCCAAAACAAGTCGGACGGCTCATTAGTTGGCCTGCTGCCCACG
CTGGCGCTGCCCGACTCCGGAATTAGTCATACCGTCTGCGAGTTACCTCCAAACGCG
AGGTTAGGCACTTGGCACTTTTCTCTGGGTCTGATGCTTTTCCAGCAGTTGTTTCTGC
TCCAGGGCTATAATTTTATCATTAAATCTGATGGCCATCATATCCCACCGATAATAG
CTGAGTGCCAATATTGCCCCAGTATCAGTAGGAAACTGCGACTTTCCTGTGCTCAAG
ATAATTTCCAAAACATAAGGTTATAGTCAGTGTAATGCGTTTAGTGATTTTTAAACA
TCTGGTTGAACACGTAATTGATTTGAATTTATATCTCACAGCAGTTCTAGGATAGAG
ATTGTGTAACAAACGGACTGGGCGGTACCTCAGATTTTAAGAAGGTATAACATTCTT
AAAATCCGTTTTCTATTTCTATGTTGTGCTATTAAATGTGGTAGTGATTTAACAACAA
ACATAATCAAAATAAATTGAGTAGTTCTACATTAAAACACCATGACTCGTAAAGTTA
TTTTTATACCCTACCAAATGAGAATGATAATTTCATCAGATTAATTAATTGTTATTTG
AATGCGGCTTGGCAACACTTTGTTGAATATACTGTTCATTTTGCATAGTGCACTAATA
CACTCAATATTTATTAATTATACATAATTTAACGACATAAAAACAGCCATGCTAGTA
AACTGCATATGGGAGAAACATAAAATTTAAAGCAACTTTGAACTCGATGAGAGTCA
TGTTTGTGTAGCGTGTTCACAAAGTCAAGGTAACCGATAAACTTCGACTTCAGTTAA
TTAATTTAATGGGTTGTGCTAATTTCGGGCTCTTAAGACAGGCAGCGCCTCCTGGCC
AGGCCCAAAAGACCTGGTGCCTGGCCAACAGATCTCCCAGTTGTCTGTCTAGAACAT
TTAGTGCCGCCAATTCGACTGAAAAACCCCTGGCAGACAACGAAAAACTTTGCTGC
GCTCGTAAAATGAAGTTGTAAAATGTATTTTTGGTATATTTCGCGGACTCATACATTC
TGGCCGATTTTGTTTTCGATTTCGTTTCAAATGGCTCGTTTGCCGGCTTCCAGCTGGA
GAGCGTAGTTTGCATTTTATTATTTCCCATATTTGAGAAATGAGCTCGGGAGCAGCG
CTTGGCAGCTTTGACAGGTCATTTGGTTGGATAATTTACGAGATTTCCAGTTAATATG
TTGGCATTACTGTTAATACTTATCCGCCGGACTTTCCGGCCGAGCTCGCCCGG

42D04_C-GAL4 (5'-3') Gibson Tails for cloning into pBPGUw digested with FseI and AatII

AAAAGTGCCACCTGACGTCACGTTATCTGTTATAAGTTATTGTTTGGCATCAATAAA
AAATTTCAAAATGTTCAAACAAAAAAGGAATAGTTTTTTATGTTTATAATTCAGTT
AATTCAGATTATGGAGTTACTGTTGGCCTTCCACCATTCGACTGCCAAAATATTCTCT
CCGTGTAGCTATAAAAAAATATCCCGAAGCTTGACCAAATAAGGCCCGCTAAGGCT
AAAAACACTTAAACAATTAGGAAACAGAGTTATTTATTTAAAGGAGGGCATTGGCT
GTAAGGAAGACATTTTCGATATCATTAATGTAATGTTAATACGGGCATTCCGTTGAC
AAGATCAACAGCAGGGGGAAGGAGGATGATTGATGCCCTGGCCCTGCTGCTTTTGTT
TGTGCGATCAAAATAATTGCTCCTTCGGGCACGATTCTTTGCCACTCAATTTATTAAA
TTTATAAAATATTTATGAAGCCGTCTCTGAGTACCTTTACTTATAAAAACTTGTTGCT

GCAAATGATGTAATTGCATGCGAAGCACTGCTTAAAACAACAATGTTGGACCCCCC
GAAAGATTGGGGCGCTGGCTCTCTGTTCCTCTGTTGCCACTTAGTAATCGGACGCCG
TCTCCGCTTGGCTTCGACTTCAGAACTGTTTACATTTCCACAGACACGCAATTTATGA
TCCGGCTTGGGGCAGGCATTGTTCGCAGCATCGGCATCGCTGCTTGCAACTGGCAAC
TGGCAACCTGCAACTTCCCTGCGGCAAATACAATACGACTGCAAGTGCGCGAAGTG
AGCGCATTCGAAACGAACTGGAATTTGGGAATGGGCAGAGCAAAGCACTTGCAGAT
TTATGGCAGGCATCTGCCCAAAGGTGCCAAGAACTTGCTTCATAAACCGAGTGTGTG
ATTGGTCTTGCCGCGGGCCAAATTGATTTATGGTCCTCTGCGGATACGTGTTAGCAG
TTATCAACTGCTAAATCAGAAGAGTTTGCGCCCGCAAATGATATTTGCCTCGTGGAA
TTGATGTCCCAATCTATGATTTCTAAGCTTGGTGGTTTAATTATAACAATAAATAACA
ATGCACTGCAGATGGGGAGCTTAAACTGTTTTAAAACGCTTACAGATGGCAAAAAA
GTGCGTTTCACACGAAGTGGGCTTAATTGATTTCTGCGTTCTGTAAATAATGCATGA
TCTATAAAACTGGGTGCTCAATTATAATAACTAATAGCTAGTCAGTGCTACTAAATA
TATATTAGAGTGCTTTAATCTGATGGCAAGTAGGGAATCAGTGCATTTCACACGAAG
ATGGTGGGTCTGCCGGCCGAGCTCGCCCGG

42D04_D-GAL4 (5'-3') Gibson Tails for cloning into pBPGUw digested with FseI and AatII

AAAAGTGCCACCTGACGTCAACACTTTGTTGAATATACTGTTCATTTTGCATAGTGC
ACTAATACACTCAATATTTATTAATTATACATAATTTAACGACATAAAAACAGCCAT
GCTAGTAAACTGCATATGGGAGAAACATAAAATTTAAAGCAACTTTGAACTCGATG
AGAGTCATGTTTGTGTAGCGTGTTCACAAAGTCAAGGTAACCGATAAACTTCGACTT
CAGTTAATTAATTTAATGGGTTGTGCTAATTTCGGGCTCTTAAGACAGGCAGCGCCT
CCTGGCCAGGCCCAAAAGACCTGGTGCCTGGCCAACAGATCTCCCAGTTGTCTGTCT
AGAACATTTAGTGCCGCCAATTCGACTGAAAAACCCCTGGCAGACAACGAAAAACT
TTGCTGCGCTCGTAAAATGAAGTTGTAAAATGTATTTTTGGTATATTTCGCGGACTCA
TACATTCTGGCCGATTTTGTTTTCGATTTCGTTTCAAATGGCTCGTTTGCCGGCTTCC
AGCTGGAGAGCGTAGTTTGCATTTTATTATTTCCCATATTTGAGAAATGAGCTCGGG
AGCAGCGCTTGGCAGCTTTGACAGGTCATTTGGTTGGATAATTTACGAGATTTCCAG
TTAATATGTTGGCATTACTGTTAATACTTATCCGCCGGACTTTGTTCGCGTTGCCCGG
CGGTCTGCTCCAAGCCGGGATTATATGCGCCCGAAATGGGGCTTAATTATATGGCCA
GAATCTATCCAGGAACAGACCACAAAAAATACTTTTCCGCACGAACAATAAAAATA
TGGTTTTCTCTTCTCGCAATTAAAGCCGTTTTTACGAGCAGAAAATTAGTGTACGAGT
AGTCGCAGCTCGCGATCTGGCCTTTGAATGGGTCTAGAATGGCTCTAAACACGCGGC
AGCTGCAGCGGATAAAGGCATAGGGCTAATAAGCTCAGGCCTTCCTTGCTGGCGAT
ATTTACAACCTGCCAAAGTCATTTGCATCGGCGTACAGGCATAATCAGCTTCCATCC
AGAAGGCATCAATTATAGGGCTCTCACCGAAGCCCAACCCCCTTTTTTTAAGCCAGG
TTCTAAGCCCCGATGTATTCACTTGAGCTGCATCCTCGAAATGCGGGCCATTAGGCT
TCGCACTCTTTTTTTTTTTGTTTTTTTTGTTTTTGACAAGTCGAAAGCTTGTGAATCCG
TGAAAACGTACTGCCCAAAAAACTAAATTACCCTGTGTGAAGATTCAACGGCTTTTT
AATTGAGTAAAATTATGGGGTACGAACAGATTTCAACAAAAAAGATGCAATGCAAG
CCGAAACACACCGGCCGAGCTCGCCCGG

42D04_E-GAL4 (5'-3') Gibson Tails for cloning into pBPGUw digested with FseI and AatII

AAAAGTGCCACCTGACGTCGGCGTTCCCAAGAACTTAGAATGGCAATACCTTAGGG
TAGCAGATATAAAAGCTGATTAGCAGCTGGTTGCCGATTCCGTCTGGGGGTGTGGAC
TCCGCCGAATCAGGTGCAATGAGGGCCATCCAACGCTCGGCTGACCACGTTCCCTAA
TTGCATCGCGGCACACAATGCGGCATACAAATGCAGCACAAACTACGACTACGACT
ACGGACAATTCCAAGACGCTCTGTCAAGCCAATCCAACATGACCATCCATCAAAGT
GCTAAGCAGGCATGAAACTTTTTCACAGTTTCATTTTCCACTCGCCGATGTCTTCTTT
ATGCATACGAGCATTATCATTATGGGCGCGTAATGGCAGCCAGACATGGATCAGAC
ATTACGGCGATCGTTTAGCAGAAGTGCAGACAATACAAACCGACGTCTGTCAACTG
CAGGCAGTTCGTCAATCAATTGAACACCGCTCCACCTCTCCCACCTCACCTAGCGCC
TCGAGCTCCTCAGTTGCCGTCGCTGGCAGCCAAGTAGCCAGGTTGTGGCCCCGATGT
CTGCAAACATGTAAACCCAAACGTAATCTCGATCGCCGCCACGTAGCTAACTAACCC
GTCTTCAAACAGGAACCGAGATCGGGTTTCCAGCCGGCCATGGCAACTTTGTTGCAA
ATTTCCTAGTCGCCCAAAACAAGTCGGACGGCTCATTAGTTTGGCCTGCTGCCCACG
CTGGCGCTGCCCGACTCCGGAATTAGTCATACCGTCTGCGAGTTACCTCCAAACGCG
AGGTTAGGCACTTGGCACTTTTCTCTGGGTCTGATGCTTTTCCAGCAGTTGTTTCTGC
TCCAGGGCTATAATTTTATCATTAAATCTGATGGCCATCATATCCCACCGATAATAG
CTGAGTGCCAATATTGCCCCAGTATCAGTAGGAAACTGCGACTTTCCTGTGCTCAAG
ATAATTTCCAAAACATAAGGTTATAGTCAGTGTAATGCGTTTAGTGATTTTTAAACA
TCTGGTTGAACACGTAATTGATTTGAATTTATATCTCACAGCAGTTCTAGGATAGAG
ATTGTGTAACAAACGGACTGGGCGGTACCTCAGATTTTAAGAAGGTATAACATTCTT
AAAATCCGTTTTCTATTTCTATGTTGTGCTATTAAATGTGGTAGTGATTTAACAACAA
ACATAATCAAAATAAATTGAGTAGTTCTACATTAAAACACCATGACTCGTAAAGTTA
TTTTTATACCCTACCAAATGAGAATGATAATTTCATCAGATTAATTAATTGTTATTTG
AATGCGGCTTGGCCGGCCGAGCTCGCCCGG
