# Supplementary material for: The yellow gene influences Drosophila male mating success through sex comb melanization
Source: eLife. 2019 Oct 15;8:e49388. doi: 10.7554/eLife.49388 (PMC6794089; doi:10.7554/eLife.49388)
Supplement: Supplementary file 4. [file elife-49388-supp4.docx]

| **Key Resources Table** | | | | |
| --- | --- | --- | --- | --- |
| **Reagent type (species) or resource** | **Designation** | **Source or reference** | **Identifiers** | **Additional information** |
| strain, strain background (*Drosophila melanogaster)* | *y^1^* | Bloomington | BDSC 169 | Backcrossed into Canton-S for six generations |
| strain, strain background (*Drosophila melanogaster)* | *Canton-S* | S. Pletcher (U Michigan) | NA | NA |
| strain, strain background (*Drosophila melanogaster)* | *UAS-yellow­-RNAi* | Vienna | KK106068 | NA |
| strain, strain background (*Drosophila melanogaster)* | *y^1^;UAS-yellow* | Bloomington | BDSC 3043 | NA |
| strain, strain background (*Drosophila melanogaster)* | *elav-GAL4* | Bloomington | BDSC 49226 | NA |
| strain, strain background (*Drosophila melanogaster)* | *nsyb-GAL4* | Bloomington | BDSC 39171 | NA |
| strain, strain background (*Drosophila melanogaster)* | *repo-GAL4* | Bloomington | BDSC 7415 | NA |
| strain, strain background (*Drosophila melanogaster)* | *dsx^GAL4^* | B. Baker (Janelia) | NA | NA |
| strain, strain background (*Drosophila melanogaster)* | *dsx^GAL4^* | S. Goodwin (Oxford) | NA | NA |
| strain, strain background (*Drosophila melanogaster)* | *fru^GAL4^* | B. Dickson (Janelia) | NA | NA |
| strain, strain background (*Drosophila melanogaster)* | *40A05-GAL4* | Bloomington | BDSC 48138 | NA |
| strain, strain background (*Drosophila melanogaster)* | *41D01-GAL4* | Bloomington | BDSC 50123 | NA |
| strain, strain background (*Drosophila melanogaster)* | *42D02-GAL4* | Bloomington | BDSC 41250 | NA |
| strain, strain background (*Drosophila melanogaster)* | *41F06-GAL4* | Bloomington | BDSC 47584 | NA |
| strain, strain background (*Drosophila melanogaster)* | *41A01-GAL4* | Bloomington | BDSC 39425 | NA |
| strain, strain background (*Drosophila melanogaster)* | *42D04-GAL4* | Bloomington | BDSC 47588 | NA |
| strain, strain background (*Drosophila melanogaster)* | *40F03-GAL4* | Bloomington | BDSC 47355 | NA |
| strain, strain background (*Drosophila melanogaster)* | *39E06-GAL4* | Bloomington | BDSC 50051 | NA |
| strain, strain background (*Drosophila melanogaster)* | *42C06-GAL4* | Bloomington | BDSC 50150 | NA |
| strain, strain background (*Drosophila melanogaster)* | *40F04-GAL4* | Bloomington | BDSC 50094 | NA |
| strain, strain background (*Drosophila melanogaster)* | *y^mCherry^* | N. Gompel (LMU Munich) | NA | NA |
| strain, strain background (*Drosophila melanogaster)* | *nsyb-GAL80* | J. Simpson (UC Santa Barbara) | NA | NA |
| strain, strain background (*Drosophila melanogaster)* | *UAS-Laccase2-RNAi* | Vienna | KK101687 | NA |
| strain, strain background (*Drosophila melanogaster)* | *dsx^GAL4-DBD^* | S. Goodwin (Oxford) | NA | NA |
| strain, strain background (*Drosophila melanogaster)* | *vGlut^dVP16-AD^* | S. Goodwin (Oxford) | NA | NA |
| strain, strain background (*Drosophila melanogaster)* | *BDSC 6993 GAL4* | S. Yamamoto (Baylor) | BDSC 6993 | NA |
| strain, strain background (*Drosophila melanogaster)* | *BDSC 49365 GAL4* | S. Yamamoto (Baylor) | BDSC 49365 | NA |
| strain, strain background (*Drosophila melanogaster)* | *BDSC 6927 GAL4* | S. Yamamoto (Baylor) | BDSC 6927 | NA |
| strain, strain background (*Drosophila melanogaster)* | *BDSC 45175 GAL4* | S. Yamamoto (Baylor) | BDSC 45175 | NA |
| strain, strain background (*Drosophila melanogaster)* | *BDSC 3740 GAL4* | S. Yamamoto (Baylor) | BDSC 3740 | NA |
| strain, strain background (*Drosophila melanogaster)* | *BDSC 5820 GAL4* | S. Yamamoto (Baylor) | BDSC 5820 | NA |
| strain, strain background (*Drosophila melanogaster)* | *BDSC 8848 GAL4* | S. Yamamoto (Baylor) | BDSC 8848 | NA |
| strain, strain background (*Drosophila melanogaster)* | *BDSC 7010 GAL4* | S. Yamamoto (Baylor) | BDSC 7010 | NA |
| strain, strain background (*Drosophila melanogaster)* | *TPH-GAL4* | S. Yamamoto (Baylor) | NA | NA |
| strain, strain background (*Drosophila melanogaster)* | *wing-body-GAL4* | Bloomington | BDSC 44373 | NA |
| strain, strain background (*Drosophila melanogaster)* | *yellow 5’ up* *EGFP reporter* | G. Kalay (UC Davis) | NA | NA |
| strain, strain background (*Drosophila melanogaster)* | *yellow intron EGFP reporter* | G. Kalay (UC Davis) | NA | NA |
| strain, strain background (*Drosophila melanogaster)* | *vasa-Cas9* | Bloomington | BDSC 51324 | NA |
| strain, strain background (*Drosophila melanogaster)* | *UAS-cytGFP* | Janelia Fly Core | NA | NA |
| strain, strain background (*Drosophila melanogaster)* | *pJFRC12-10XUAS-IVS-myr::GFP* | Janelia Fly Core | NA | NA |
| strain, strain background (*Drosophila melanogaster)* | *42D04_A-GAL4* | this paper | NA | GAL4 line with cloned sub-fragment from 42D04 doublesex enhancer sequence (see Supp. Fig. S6) |
| strain, strain background (*Drosophila melanogaster)* | *42D04_C-GAL4* | this paper | NA | GAL4 line with cloned sub-fragment from 42D04 doublesex enhancer sequence (see Supp. Fig. S6) |
| strain, strain background (*Drosophila melanogaster)* | *42D04_D-GAL4* | this paper | NA | GAL4 line with cloned sub-fragment from 42D04 doublesex enhancer sequence (see Supp. Fig. S6) |
| strain, strain background (*Drosophila melanogaster)* | *42D04_E-GAL4* | this paper | NA | GAL4 line with cloned sub-fragment from 42D04 doublesex enhancer sequence (see Supp. Fig. S6) |
| other | 35 mm diameter Petri dish | Genesee Scientific | catalog #32-103 | for mating assays |
| other | 17 inch LED light pad | HUION via Amazon.com | HUION L4S | for mating assays |
| other | Canon VIXIA HF R500 | Canon via Amazon.com | VIXIA HF R500 | for mating assays |
| other | Manfrotto tripod | Manfrotto via Amazon.com | MKCOMPACTACN-BK | for mating assays |
| other | Fascam Photron SA4 | Photron | SA4 | for high-speed mating assays |
| other | 105 mm AF Micro Nikkor Nikon | Nikon | 105 mm AF Micro Nikkor | for high-speed mating assays |
| other | 96 well cell culture plate | Corning | 05-539-200 | for high-speed mating assays |
| other | Schneider’s insect medium | Sigma | S0146 | IHC |
| antibody | rabbit polyclonal anti-GFP | ThermoFisher Scientific | A-11122 | 1: 1000 |
| antibody | mouse monoclonal anti-BRP hybridoma supernatant | Developmental Studies Hybridoma Bank, Univ. Iowa | nc82 | 1: 30 |
| antibody | Alexa Fluor® 488-conjugated goat anti-rabbit polyclonal | ThermoFisher Scientific | A-11034 | NA |
| antibody | Alexa Fluor® 568-conjugated goat anti-mouse polyclonal | ThermoFisher Scientific | A-11031 | NA |
| antibody | rabbit polyclonal anti-dsRed | Clontech | 632496 | 1: 1000 |
| antibody | rat monoclonal anti-*D*N-Cadherin | Developmental Studies Hybridoma Bank, Univ. Iowa | DN-Ex #8 | 1: 100 |
| antibody | Cy^TM^3-conjugated goat anti-rabbit polyclonal | Jackson ImmunoResearch | 111-165-144 | NA |
| antibody | Cy^TM^5-conjugated goat anti-rat polyclonal | Jackson ImmunoResearch | 112-175-167 | NA |
| commercial assay or kit | MEGAscript T7 Transcription Kit | Invitrogen | AM1334 | in vitro transcribing gRNAs to delete the *yellow* MRS sequence: GCAGTTTTAAATGTCGATGA and GATTACCCGAACACTGAACC |
| recombinant DNA reagent | One Shot TOP10 DH5alpha competent cells | Invitrogen | C404006 | for cloning homology arms into the 3xp3-DsRed donor plasmid |
| recombinant DNA reagent | pHD-DsRed-attp | Addgene | Plasmid # 51019 | for cloning homology arms into the 3xp3-DsRed donor plasmid |
| recombinant DNA reagent | forward primer with NcoI tail for cloning homology arm immediately flanking the *yellow* MRS sequence on the 5’ | this paper | IDT | 5’-TTACCATGGGGGATCAAGTTGAACCAC-3’ |
| recombinant DNA reagent | reverse primer with BglII tail for cloning homology arm immediately flanking the *yellow* MRS sequence on the 5’ | this paper | IDT | 5’-GGAGATCTGGCCTTCATCGACATTTA-3’ |
| recombinant DNA reagent | forward primer with Bsu36I tail for cloning homology arm immediately flanking the *yellow* MRS sequence on the 3’ | this paper | IDT | 5’-TACATCCCTAAGGCCTGATTACCCGAACACT-3’ |
| recombinant DNA reagent | reverse primer with MluI tail for cloning homology arm immediately flanking the *yellow* MRS sequence on the 3’ | this paper | IDT | 5’-TATACGCGTTGCCATGCTATTGGCTTC-3’ |
| recombinant DNA reagent | restriction enzyme NcoI | NEB | R0193S | for cloning homology arms into the 3xp3-DsRed donor plasmid |
| recombinant DNA reagent | restriction enzyme BglII | NEB | R0144S | for cloning homology arms into the 3xp3-DsRed donor plasmid |
| recombinant DNA reagent | restriction enzyme Bsu36I | NEB | R0524S | for cloning homology arms into the 3xp3-DsRed donor plasmid |
| recombinant DNA reagent | restriction enzyme MluI | NEB | R0198S | for cloning homology arms into the 3xp3-DsRed donor plasmid |
| sequence-based reagent | forward primer used to confirm *yellow* MRS sequence deletion | this paper | IDT | 5’- CAGTCGCCGATAAAGATGAACACTG-3’ |
| sequence-based reagent | reverse primer used to confirm *yellow* MRS sequence deletion | this paper | IDT | 5’- CAAGGTGATCAGGGTCACAAGGATC-3’ |
| recombinant DNA reagent | IDT gene blocks for cloning 42D04-GAL4 enhancer sub-fragment lines | this paper | IDT | see Supplementary File S1 |
| recombinant DNA reagent | pBPGUw GAL4 plasmid | Addgene | Plasmid #17575 | for cloning 42D04-GAL4 enhancer sub-fragment lines |
| recombinant DNA reagent | restriction enzyme FseI | NEB | R0588S | for cloning 42D04-GAL4 enhancer sub-fragment lines |
| recombinant DNA reagent | restriction enzyme AatII | NEB | R0117S | for cloning 42D04-GAL4 enhancer sub-fragment lines |
| recombinant DNA reagent | Mix and Go! DH5 alpha competent cells | Zymo | T3007 | for cloning 42D04-GAL4 enhancer sub-fragment lines |
